# Implementation lessons learned from the University of California’s Diabetes Prevention Program Initiative

**DOI:** 10.1186/s12889-024-20198-1

**Published:** 2024-10-11

**Authors:** Tamra Burns Loeb, Kate Ramm, Maryam Gholami, Kelly Shedd, Samantha Soetenga, Meera Bhagat, Nicholas J. Jackson, Un Young Rebecca Chung, O. Kenrik Duru, Carol M. Mangione, Alison B. Hamilton, Tannaz Moin

**Affiliations:** 1 David Geffen School of Medicine at the University of California, Los Angeles, CA USA; 2 Altman Clinical and Translational Research Institute (ACTRI), University of California, San Diego, USA; 3 University of California, Irvine, USA; 4 Campus Recreation, University of California, Los Angeles, CA USA; 5 VA Greater Los Angeles Healthcare System, Los Angeles, CA USA

**Keywords:** Diabetes prevention program, University-based, Implementation partners, Factors influencing success

## Abstract

**Background:**

The University of California’s Diabetes Prevention Program (UC DPP) Initiative was implemented systemwide to address diabetes and obesity risk on all 10 campuses. As little is known about implementing lifestyle change programs in university settings, we examined implementation partners’ (i.e., UC DPP leaders and campus leads) perceptions of factors influencing program success on UC campuses.

**Methods:**

We conducted qualitative interviews with UC DPP leaders and campus leads to examine challenges and opportunities with university-based DPP delivery models. Interviews were recorded, professionally transcribed, and reviewed in detail by the research team. Transcripts were analyzed using rapid qualitative analysis (RQA). The study was approved by the UCLA Institutional Review Board. All implementation partners provided verbal informed consent.

**Results:**

Twenty-six implementation partners (8 UC DPP leaders and 18 campus leads) completed interviews in 2021. Seven themes were identified as critical for implementation, including (1) marketing and recruitment (i.e., market and recruit broadly through established channels as well as target at-risk populations); (2) enrollment (i.e., offer the program during convenient times and let participants know what to expect); (3) use an adaptable, evidence-based program; (4) secure funding for the program, participants, lifestyle coaches, and space; (5) hire experienced and dedicated staff and lifestyle coaches; (6) ensure leadership support; and (7) utilize campus linkages and resources. Perceptions of challenges faced with respect to these themes are also described.

**Conclusions:**

This is one of the first studies to examine the challenges and opportunities of delivering an intensive lifestyle change program across 10 university sites. Understanding factors that enhance success of university-based diabetes prevention programs can facilitate UC DPP efforts and help inform delivery strategies of health and wellness programs across other university settings more broadly.

**Supplementary Information:**

The online version contains supplementary material available at 10.1186/s12889-024-20198-1.

## Background

Prediabetes affects 38% of U.S. adults and increases risk of incident type 2 diabetes, a leading cause of morbidity, mortality and healthcare costs in the U.S. ([1]; https://www.cdc.gov/diabetes-prevention/about-prediabetes-type-2/index.html). The Diabetes Prevention Program (DPP) is a year-long intensive lifestyle intervention shown to lower type 2 diabetes risk among at-risk adults ([2]; https://www.cdc.gov/diabetes-prevention/lifestyle-change-program/index.html). Over the past two decades, substantial research has been conducted on implementing the DPP in a variety of health facility, employer, and community settings [3–5]. However, few studies examining university- or campus-based approaches for delivery of intensive lifestyle interventions have been published [6–9]. 

A recent systematic review and meta-analysis concluded that offering DPP interventions in the workplace is an effective strategy to prevent type 2 diabetes [10]. Research also demonstrates that college students with known risk factors (e.g., overweight/obesity status, sedentary lifestyle, family history) tend to underestimate their risk for developing type 2 diabetes and could benefit from diabetes education [11]. University students, staff and faculty are often offered access to a variety of university resources, making university campuses an ideal setting for implementation of DPP [12]. However, despite research demonstrating variation in lifestyle behavior change program content and duration (i.e., [13]), and facilitators and barriers to participant engagement in healthy lifestyle behaviors (i.e., [14]), little is known about factors that influence the successful implementation of university-based DPP programs.

In 2018, the University of California (UC) implemented the DPP across all 10 UC campuses to augment obesity and diabetes prevention efforts aimed primarily at staff and faculty. All 10 UC DPP sites adhere to and are recognized by the Centers for Disease Control and National Diabetes Prevention Program [6], (https://www.cdc.gov/diabetes-prevention/php/program-provider/program-requirements.html; https://www.cdc.gov/diabetes-prevention/lifestyle-change-program/index.html). The first UC campus to implement DPP was the University of California, Los Angeles (UCLA). The UC DPP Coordinating Center, which provides technical assistance, logistics, and coordination to individual campuses, is housed at UCLA.

UC DPP implementation partners include UC DPP leaders and campus leads. UC DPP leaders hold leadership positions across campuses or at the UC Office of the President and provide support for UC DPP implementation across multiple campuses. Campus leads are coordinators, coaches, and administrators who manage day-to-day UC DPP-related activities on a specific campus. The UC DPP is offered free of charge to all UC affiliates (e.g., faculty, staff, and students) who meet National DPP eligibility criteria (https://www.cdc.gov/diabetes-prevention/php/program-provider/program-requirements.html).

To address gaps in our understanding of what factors influence the success of UC DPP on university campuses, we asked UC DPP implementation partners about their experiences. The perceptions of UC DPP implementation partners can guide future efforts to enhance the successful implementation of DPP on college and university campuses.

## Methods

Guided by the RE-AIM framework [15], we developed two semi-structured interview guides, with separate versions tailored to UC DPP leaders and campus leads (For a Summary, see [8], See Appendices 1 and 2 for complete interview guides). The interview guides assessed 7 domains, including general questions (i.e., What is your role at [insert campus name] campus and How did you become involved with UC DPP?), Reach (i.e., What recruitment strategies has your campus used?), Effectiveness (i.e., How do you evaluate the success of DPP on your campus?; How effective would you say UC DPP has been on your campus?), Adoption (i.e., What are facilitators to UC DPP adoption on your campus?; What are barriers to UC DPP adoption on your campus?), Implementation (i.e., Can you describe the UC DPP implementation process on your campus?) and Maintenance (i.e., To what extent do you anticipate UC DPP will become established in your campus culture and workflow?; What are the obstacles to sustaining UC DPP on your campus over time?). We also included several questions about the challenges related to COVID-19 pandemic campus closures (i.e., How do you think COVID has impacted DPP on your campus?). UC DPP leader and campus lead interview guides were similar, with campus leads describing factors influencing success on their campus and UC DPP leaders answering questions with respect to the UC system (i.e., across all campuses).

We sent emails and letters to UC DPP implementation partners to invite them to participate in our interviews. Interviews were conducted by a team member trained in qualitative research methods over UC Zoom and lasted approximately 1 hour.

### Qualitative data collection

Between April and August 2021, we conducted in-depth qualitative interviews to better understand UC DPP implementation partners’ perceptions and experiences with the UC DPP [6, 16]. Interviews were recorded, professionally transcribed, and transcripts were reviewed in detail by the research team. After familiarization with the transcripts, we used Rapid Qualitative Analysis (RQA) as our analytic approach. RQA is an “action-oriented approach” (St. George 2023:1) to qualitative data analysis that enables researchers to analyze results quickly to inform ongoing practices with at-risk populations [17–19]. This approach facilitates rapid identification or expansion of knowledge of intervention components as well as challenges and opportunities of a program [15, 18, 20]. The research team adhered to a well-delineated, established RQA approach developed by Hamilton and colleagues, which has been utilized in numerous health equity projects and health services research studies [17–19, 21], as well as by our team see [8]. Responses to questions were reviewed by team members. For each transcript, a templated summary was created to inventory the implementation partners’ responses to each of the respective interview guide domains. Team members (T.B.L., K.S., S.S., T.M., A.H., M.B., and O.K.D.) first summarized the same two transcripts and discussed until consensus was achieved to establish inter-rater reliability. These summaries were then combined by two team members (T.B.L. and K.R) into matrices for identification and comparison of themes related to challenges and opportunities with each campus-based DPP delivery model across all RE-AIM dimensions, as well as to establish thematic saturation (i.e., sufficient, cross-cutting evidence for themes that are presented below, [18, 22]. The study was approved by the UCLA Institutional Review Board. All implementation partners provided verbal informed consent and were offered a $50 gift card.

## Results

Twenty-six implementation partners (8 UC DPP leaders and 18 campus leads) completed interviews. Seven themes related to a university-based DPP were identified across implementation partners, including (1) marketing and recruitment; (2) enrollment; (3) retention; (4) funding; (5) leadership support; (6) linkages and resources; and (7) staff and lifestyle coaches (See Table 1). While some of the themes are interrelated (e.g., financial resources impact recruitment strategies and ability to hire coaches), we describe each in more detail below and indicate the corresponding RE-AIM domain.

**Table 1:** Themes, opportunities, and challenges associated with UC DPP

**Theme**	**Opportunities**	**Challenges**
Marketing and recruitment	• Market and recruit broadly through established communication channels ◦ System-wide and campus-specific communication ◦ In-person marketing strategies ◦ Word of mouth • Target at-risk populations ◦ Market to providers ◦ Diabetes-specific website blasts, events, and outreach to subpopulations	• Smaller campuses have fewer communication channels ◦ Lack “overall awareness”, “branding and promotion” “standardized approach to recruitment,” and higher level of communication • Need improved marketing and recruitment of high-risk populations • Confidentiality concerns regarding collection of health information
Enrollment	• Offer the program on work time, let participants know what to expect • Hold information, orientation sessions • Supervisors provide release time	• Incompatibility of sessions with participant schedules • Some supervisors reluctant to give release time
Retention	• Modify and/or enhance the curriculum ◦ Add sessions, hold office hours, make up sessions, provide supplementary materials, sessions, individual counseling ◦ Tailor for university setting ◦ Support group post-program ◦ Add small incentives, utilize social media, group walks or events, enlist guest speakers ◦ provide resources, reminders	• Lack of finances to: ◦ hire supplemental staff to enhance curriculum ◦ tailor program to participants ◦ offer additional incentives for participation
Funding	• Secure funding for participants, lifestyle coaches, and space • Offer the program for free, during the workday, provide free access to space	• Financial support needed for program materials, to hire coaches, Spanish-speaking health educators, administrators, and pay for space
Leadership support	• Ensure leadership support within the UC system and on individual campuses • Coordinating Center that conducts systemwide calls, provides support, expertise, lifestyle coach training	(Partners did not identify leadership support challenges)
Linkages and resources	• Utilize campus wellness linkages and other resources • Offer the program under a wellness department/focus • Internal program • Campus medical center • Collaboration across campus • Coaches knowledgeable about campus culture, food, and resources	• Smaller campuses, those with that were not fitness or wellness oriented • Lack of collaboration between health system and campus recreation or wellness coordinator ◦ Limited access to rooms or times • Difficulty identifying where UC DPP fit in the “recreation portfolio” • Competing priorities
Staff and lifestyle coaches	• Hire experienced and dedicated staff and lifestyle coaches ◦ Coaches from School of Medicine ◦ Bilingual educators	• Program coordinator, administrator are often a single position • Challenges with recruiting, hiring, training, and paying coaches • Lifestyle coach retention

### Theme 1: marketing and recruitment

System-wide and campus-specific marketing and recruitment were perceived as critical factors for program success by facilitating reach. Implementation partners stressed the importance of both casting a wide participant recruitment net and targeting at-risk campus populations using established, internal channels of communication to promote UC DPP.

UC DPP leaders stressed that UC DPP was advertised as an internal program to staff and faculty (e.g., through employee and campus listservs, benefit partners, newsletters, flyers, and mailers). One UC DPP leader noted, “…we were able to get access to our UC Health’s electronic medical records across the system, and we used that information to identify potential candidates for the program. And letters were sent to the—I think still today, letters are sent to their homes. The campuses themselves also do marketing of the program. And we on my team,… every year we send out a mailing to all of our employees that are covered on our health plans to remind them or to introduce them to the systemwide program.”

Marketing strategies that were noted by campus leads on specific campuses included providing promotional materials to wellness ambassadors, distributing flyers at pre-shift employee meetings, sharing visual information on workplace monitors (e.g., when campus employees swipe in for shifts), discussing UC DPP in Healthy Campus initiative-related activities, advertising in staff assemblies, leading physical activities (e.g., a stretch) in campus Town Halls and having attendees complete prediabetes screeners, and recruiting at benefits fairs and campus-specific events. In addition, campus leaders capitalized on UC DPP participants to spread the word to their colleagues. Students on some campuses were recruited through the Student Health Center.

#### Challenges to broad marketing and recruitment

Campus leads described the limitations of these usual outlets; one expressed concern that their mailing lists were saturated: “in some ways the program is challenging to keep it fresh, keep people interested; [I] think [the program] will hit a threshold where everyone has been through a cohort.” Smaller campuses also reported having fewer communication and marketing channels. Some campus leads noted that “overall awareness” of UC DPP, as well as “branding and promotion” needed improvement. Others wanted a “standardized approach to recruitment,” as “there’s not a good, coordinated way to get the word out to everyone, it’s hodgepodge but we try our best.” Several noted that they lacked leaders to promote the program, wanted a “higher level of communication, like a chancellor’s email,” and wished the “communication chain could be promoted from OP (Office of the President), to send out a campus-wide email” so that there was “support that trickles down to the employee level.” Campus leads also expressed concern that many employees fail to open emails that contain UC DPP information.

#### Target at-risk populations

Implementation partners also emphasized the importance of specifically targeting higher-risk populations. According to one UC DPP leader, “Initially… [we] compiled a broad-based recruitment of individuals, and then targeted key departments that represented a workforce segment… “ Some campuses distributed UC DPP flyers to providers in occupational health or in campus-affiliated health care systems to act as champions by displaying UC DPP materials in their clinic areas. One campus lead described their use of a website with a “Do You Have Prediabetes?” link that was blasted when new cohorts were being recruited. Another stated, “we did a ‘Know Your Numbers’ event, so we did blood lipids, glucose, to drum up awareness of what prediabetes is, [and] we highlight diabetes and prediabetes in the wellness ambassador newsletter.” For these events, there were health education consultants on hand to recommend UC DPP to individuals who qualified. Finally, some campus leaders targeted specific campus subpopulations, including Housing, Dining and Auxiliary Services.

#### Challenges to reaching at-risk populations

Other implementation partners described wanting to improve reach to at-risk groups, and difficulties with accessing high-risk campus populations. Implementation partners noted that men have been underrepresented in UC DPP cohorts across the UC system, and one UC DPP leader voiced that efforts are needed to increase outreach to male students, staff and faculty. Others voiced the desire to target student populations, as efforts to recruit were primarily aimed to staff and faculty. Some campus leads described ineffective or scant marketing and recruitment targeted to specific racial/ethnic groups, and that Spanish-speaking groups on campus have been “underserved.” One campus lead indicated that their campus could do a better job of promoting to staff and faculty, as well as student primary care providers in the health system as well as through Student Health.

Confidentiality concerns were a frequently reported challenge. One UC DPP leader stated, “I think there’s always a concern with confidentiality. I think people worry about who’s going to know my data, who’s going to know about my health information.” Similarly, a campus lead described a “hesitation because the program is associated with the university… (employees may be) scared to have (their) employer involved in personal health journey.”

### Theme 2: enrollment

Several factors that enhanced reach by facilitating enrollment efforts were perceived as critical for UC DPP success.

#### Offer the program on work time and let participants know what to expect

The capacity to offer employees to participate in the program on work time, or during a lunch hour or early evening after work, was perceived as a critical enrollment facilitator. Participant convenience and accessibility was frequently mentioned by UC DPP leaders: “the easier you make it, the more uptake there is”; “make sure the program is “easy for campus people.” Others stated, “… my awareness of how the program is set up, it’s made to be very accessible”; “…if you make it easy for people to go at lunch or some convenient time for them and they don’t have to travel very far or take too much time out of their day, I think that’s really the biggest thing.” One campus leader stressed how important it is to ‘make it easy to get to.”

A second enrollment facilitator involved holding information and/or orientation sessions (sometimes referred to as “session zero”). Campus leaders emphasized holding “a lot of info sessions leading up to the actual launch of the program to gain interest,” and holding a “one-on-one meeting before the first session.” Another noted that there was “experience in my department with the program and with motivational interviewing... good infrastructure, in terms of enrolling people.”

#### Challenges to enrollment

Implementation partners described challenges due to the incompatibility of sessions with participant schedules. For instance, it can be difficult for those that work night shifts on campus to meet for an hour. One UC DPP leader stated, “For some people, it’s not convenient because they can’t just take an hour off of work, especially employees that are hourly, also people realize this is a pretty intensive lifestyle change program and that it’s over a year long, and that commitment makes some people uncomfortable.” Several campus leaders noted that Housing and Dining campus employees had less flexibility in terms of their schedules, as they are represented by a collective bargaining agreement; however, one noted that “a couple higher ups got excited about offering it housing and dining staff and made it happen on paid time.” Others reported that some supervisors were reluctant to giving release time for participants to attend sessions.

### Theme 3: use of an adaptable, evidence-based program

Implementation partners were enthusiastic about DPP being evidence-based, facilitating program effectiveness. One UC DPP leader noted, “The recognition that the CDC-approved diabetes prevention program, in essence, can prevent or delay the onset of diabetes, and that’s a positive from a wellness point of view.” According to another, “I think the most impressing part is the comprehensive holistic approach that the program takes. And I think that’s something—and very thoughtful, as well, and the evidence-based structure behind it which is really what we are trying to disseminate across the campuses. But one could look at it as the gold standard of a type of a program like this… I do think that it really is the type of a program that we are trying to disseminate and advance across the campuses.” Campus leads also described trusting the program due to its evidence base: “And so having this evidence-based CDC program that all the UCs were doing was just a great way to get involved with a program that was already in existence.”

#### Campus leads also discussed enhancing the DPP curriculum and/or sessions to mitigate against attrition

One campus lead discussed adding sessions when the curriculum transitioned to monthly meetings to sustain engagement; “we have a lot more retention when we meet more often.” Another described talking “more about the benefits, the nutritional, as opposed to the weight loss being the focus.” Some campus leads held office hours and make up sessions for participants, hired a Registered Dietician to add supplementary materials, and had a nutritionist available for sessions or individual counseling. Some discussed “upgrading” the curriculum to target individuals with a higher educational level (e.g., more detailed and expanded curriculum regarding nutrition, weight loss, and physical activity), adding a support group post-program, including teaching kitchen classes, and having personal trainers or nutritionists lead some sessions. Some campus leads added small incentives (e.g., raffle, organizing outings) or fun games to keep participants engaged, utilized social media, added a group walk or stretch session, and enlisted guest speakers (e.g., a wellness program specialist). Another created a “calendar of events of what’s going on on campus, other resources, and we would promote all our other programs going on, just to stay in touch with them.” Some added additional time slots and locations based on volume of participants, and provided reminders, including personal follow-ups (phone calls and email reminders).

#### Challenges with evidence-based programs or adaptability

One campus lead described the “need to adapt programs or offer slightly different things to make them new and exciting; it’s hard to do that with an evidence-based program.” Campus leads also described specific challenges with respect to enhancing the curriculum. Some campuses lacked the finances to hire supplemental staff to better tailor the program to participants (e.g., chef demonstrations, dieticians, and personal trainers to lead stretches), or to offer additional incentives for participation.

### Theme 4: funding

Implementation partners described the role of funding as critical for program adoption, implementation, and maintenance.

#### Secure funding for the program (i.e., participants, lifestyle coaches, and space)

UC DPP leaders stressed the critical role of funding and in-kind resources from the University of California system in implementing and maintaining UC DPP. One noted it “would not have happened without the funding,” and another stated that it was critical to have “the funding to get us started.” Another UC DPP leader stated, “I had been asking to start a DPP since 2015, … was on my radar but couldn’t get traction because budget was a barrier, so when the Office of the President offered funding, the barriers were removed, and we were green lighted.” According to another, “In the beginning, we didn’t have a consistent way that we communicated with campuses. I mean, we overcame that. We started these monthly meetings which your funding, the campus’s funding would be contingent on attending these monthly meetings. And they were reporting out financials for their program every so often, every quarter, I think. So, I think that helped to have that structure there. And so clear requirements and expectations for the campuses to participate and receive the funding.”

Implementation partners also attributed the success of the program to being able to offer it to participants free of charge through UC funding. The fact that the program was a free campus resource and at several campuses was offered during paid work time was perceived as critical to its success. One UC DPP leader noted, “I would say however the finances are…I think each campus should support it. And most of the time, each campus has to come up with their own resources, but I’m sure there’s some central funding, but I don’t know that aspect of it. Let’s just say, yes, I think UC as a whole in some way should sustain it…” Another UC DPP leader stated, “that the campuses didn’t really have to pay anything because we had funded it. So I think that also helped.”

Campus leads also described the importance of the program being offered free of charge to its success; one voiced, “It’s free and they know their employer is committed to helping them improve their health, stay well, and supporting them, sending them the message that we’re here for them.” Campus leads worked with supervisors who were willing to allow their employees to attend sessions for an hour during their shifts, typically during the lunch period. UC funding was used to support UC DPP lifestyle coach salaries, via hiring new part-time coaches or utilizing current part-time staff (personal trainers, RDs, etc.) who completed DPP coach training. Another key factor was in-kind contributions from individual sites, including access to campus space without fees; according to one campus lead, “we were able to book rooms at no cost,” and another noted, “And even when we were doing it in person…I didn’t charge for the space and there was support there to reserve a conference room every week for a certain time so we could have these classes there.”

#### Challenges related to funding

Many UC DPP leaders felt the lack of consistent year after year funding led to uncertainty about long-term sustainability of the program. According to one, “There is an ongoing looking for sustainability in funding, a program that is this mature and has showed positive results, you would hope to be able to find something that’s a little bit more sustainable, so that the focus can be continuing to improve upon the program and not the funding.” Another noted that some “departments are worried about release time and how they pay for it.”

Many campus leads perceived program materials to be a financial burden (e.g., printing the flyers, curriculum, and binders were expensive and time consuming to assemble). Some campuses had to secure additional funding to make copies to hand out to participants. One noted, “We printed all the curriculum and put them into a binder and it was a huge cost and too much work, so we emailed future cohorts the curriculum on a weekly basis, my wellness program specialist sent out the weekly curriculum in advance, we experimented with sending it the day before, or an hour before (enough time to make sure you got it but not enough time to read it and not go to the session).”

Some campuses lacked the financial means to hire coaches and ensure staff stability. Campus leads frequently reported that financial support was needed for hiring a “dedicated facilitator,” as hiring consultants and vendors was perceived to be difficult and take too long. Several campus leads reported experiencing difficulties paying coaches. Others couldn’t offer additional classes, needed to accommodate specific subpopulations, due to a lack of staff. For example, one campus lead stated, “…We had people who wanted to join from Dining or Housing, and our in-person classes were always at noon, which is a busy time for Dining, they couldn’t always get off to join. We had limited staff and couldn’t offer classes throughout the day”.

Another campus lead noted, “How do we pay the coaches? They were employees, and this was either extra hours after their shift and not in the cost center they worked out of so we had to pick an HR cost center and they clocked in and out of that time center for the time they spent with DPP, there was pushback from HR about that.” Others needed funds to hire Spanish-speaking health educators so that they could offer the program in Spanish.

Several campus leads described financial challenges regarding having to pay for room reservations for sessions. Others noted that scheduling sessions was difficult due to a lack of time and funding for hiring administrators.

### Theme 5: staff and lifestyle coaches

Lifestyle coaches were perceived as playing an important role in the implementation success of UC DPP. According to one UC DPP leader, “it’s homegrown, not an external vendor coming in and conducting the program, developed at UC from the ground up, run and operated by UC staff,” and “…the best example because they are invested in this for their staff and have seen data and feedback that is amazing.” Campus leaders provided specific information about desired staff and lifestyle coach characteristics.

#### Hire experienced and dedicated staff and lifestyle coaches

Implementation partners stressed the importance of having well-qualified and experienced UC DPP staff and lifestyle coaches, often from the School of Medicine or other health professions. Campuses that had a “good veteran coach” with a broad or diverse set of skills were perceived as critical for program success. One campus lead described their campus as having great coaches from the School of Medicine who were already trained as health coaches and had excellent motivational interviewing skills; they were “all health majors, pre-med, so it was easy, they didn’t need to be trained on how to coach, only needed to know the logistics of the DPP program.” Campus leads noted that the success of a given cohort was dependent on the coach’s skills (i.e., they were engaging, knew when to be silent and how to encourage participation).

One campus lead stated, “We have a lot of bilingual educators (Spanish, Chinese, other languages). I am giving the opportunity for everybody, served or underserved, to participate. I know some of the people that are participating are in the underserved communities.” Another noted that they were lucky to have had their lifestyle coach and felt this was major strength: “Because I know other UCs struggle finding instructors and someone to take this on, but we had the bandwidth and the space, and this is something I’ve been wanting to do for a long time.”

#### Challenges related to staff and lifestyle coaches

Some campus leads described obstacles related to the program coordinator and administrator being a single position. Many noted that an additional individual is required to do the data collection as the data is “overwhelming to collect.” According to one campus lead, “What’s been really helpful for me is to have a lifestyle coach that can do some of the data collection… And so, I think, as long as we can have a coordinator and a really, really good lifestyle coach, we’ll be just fine.”

Numerous challenges with respect to identifying, hiring, training, and paying coaches were described by campus leads. Campus leads reported problems with the recruiting and hiring process; depending on how the coaches were hired (i.e., as a contractor), it could take a very long time. Some campuses struggled with training students to be effective group facilitators and trying to match facilitators to the needs of specific cohorts (e.g., age group, Spanish-speaking, participant goals).

Another frequently reported issue was difficulty with lifestyle coach retention. Campus leads described positions being eliminated, and coaches that didn’t show up, leading to participant attrition. Sometimes this was attributed to not having someone to “champion” the program. For example, if not included in one’s job description, the program became an “added project.” Some campus leads indicated that their coaches lacked diversity in backgrounds, languages spoken, and ability to work with specific subpopulations. They also wished that they had lifestyle coaches with campus-specific knowledge, who were connected to campus culture and resources.

### Theme 6: leadership support

#### Ensure leadership support

Implementation partners also talked about the importance of leadership support within the UC System and on individual campuses for program adoption and implementation. UC campus leadership support of the UC DPP was perceived as critical for the program’s success, primarily through promoting collaboration between the campuses. One UC DPP leader noted, “So we have real relationships with all different stakeholders on campus that allowed us to connect…We identified all the people that this would be an important activity for them to be involved in and we brought them together, and they all left their personal agenda at the door and agreed to do it, and set up different measures of success, and I think that’s why it still continues.” UC DPP leaders also described the importance of a strong UC-wide leadership team, including support from UCOP, HR, and UC Health. One stated, “We have a really, really, good strong, top-down support that some of the campuses have really struggled with…We’re very, very lucky to have some higher stakeholders who are huge proponents of the program.” Another stated, “…I would say strong project and program management was probably the most important thing we learned. And active management and handholding I think in the beginning for campuses as they’re starting the program because there’s a lot of handholding that we needed to do and that you need to do to keep the campuses engaged.” According to another UC DPP leader, “It takes a village, [and] everybody has to start somewhere…I would most definitely recommend talk to others that have been involved…You need top-down support, but you need bottom-up energy. So, get the right players in the room, the right people in the right seats of the bus, that’s what’s going to make the whole bus roll…. You’ve got to build support, build a community, pilot it somewhere, let the buzz be positive, and then let it spiral from there.”

Campus leads also discussed the importance of securing leadership buy-in and having a strong leadership team, “there’s a large leadership core that understand it and believe [in] it…, so I feel that we have the opportunity to make it, and we’re the ones who are driving it at the UC Office of the President. So we continue to do that and I think it’s really important.” Another campus lead voiced that “leadership at the Chancellor’s level has showed support of wellness and funding that and by continuing to fund.”

Many implementation partners also noted the importance of the UC DPP Coordinating Center to program success. According to one UC DPP leader, it is “helpful that all the campuses have dedicated wellness representatives or wellness departments or a wellness focus, also managed by a very strong team….the [UC DPP] Coordinating Center team is very strong.”

Campus leads reported feeling connected with the Coordinating Center and being supported with “tools and information.” Other strengths of the UC DPP Coordinating Center described by campus leads included being responsive, “incredibly helpful,” and always asking campuses what they needed to be successful: “Having it be part of a systemwide effort, having the expertise of the [UC DPP] Coordinating Center to provide us with the technical assistance, and logistics, and coordination. Data collection was helpful. And then at [XX], diabetes was also a high-risk area and a need.” The lifestyle coach training, led by the [UC DPP] Coordinating Center, was described as “intensive,” “very thorough,” and “fantastic,” and the systemwide calls were extremely informative; both were perceived as contributing to program success. 

### Theme 7: linkages and resources

Program adoption and implementation were influenced by campus linkages and resources.

#### Utilize campus wellness linkages and other resources

The ability to offer UC DPP under a “wellness umbrella,” that is, having a wellness department, representative, or focus was also frequently mentioned as important for program success. According to one UC DPP leader, “I think the wellness coordinators at the campuses, the wellness team at the campuses, they really believed that the program could work because we were showing them or communicating to them all the evidence, the research that had been done to support the structure of the program and success of the program.” Another UC DPP leader stated, “I think because it was introduced in our platform of the Healthy Campus Initiative, we already had key stakeholders at the table… being able to say, here’s an amazing opportunity and we already have all the components on the table I think is an accelerator… We have expertise, we have spaces, we have great researchers, we have a willing audience.” Other UC DPP leaders noted that the program is a “good extension of what we currently do,” and “We have a wide range of fitness and stress reduction and some things you would find in a recreation department. But DPP because it’s more specific to a chronic illness was unique. It was a unique offering and opportunity for us to do something slightly different.”

Campus leads also stressed the value of having a campus medical center. Campuses with medical centers were able to recruit lifestyle coaches from the school of medicine; at one campus, the Healthy Campus champion was the Dean of the School of Medicine. If there was a lack of connection with a health system, partners reported slower uptake of DPP. Additionally, campus leads emphasized that the department that offered the program mattered; advantages were noted with respect to being able to partner with a wellness program, campus health system, or associated medical center. Situating DPP in departments housed under “wellness” or recreation had greater reach and were perceived as more effective.

Having campus linkages allowed some campuses to collaborate with professionals from across campus (i.e., psychologists, physical therapists, occupational therapists, and clinical nutrition students) for some sessions. Campus leads also described how helpful it is for coaches to be “connected,” know the “culture, foods on campus,” and resources available on a given campus.

#### Challenges related to lack of linkages and/or resources

According to implementation partners, campuses that were smaller, had a “separation” between groups leading wellness efforts across the campus (e.g., not led by the same individual), or not “fitness or wellness-oriented” struggled. A lack of collaboration between the health system and campus recreation or the wellness coordinator was perceived as detrimental to establishing a network for referrals and ensuring there was adequate bandwidth to sustain a successful program.

Another frequently reported challenge was finding a consistent space throughout the year for people to meet in a central campus location. Some campus leads reported that locations were difficult to secure because of the coordination required to hold groups before and after work hours; this limited access to rooms and times.

#### Other linkage-related challenges

Other challenges included identifying where UC DPP fit in the “recreation portfolio.” For example, campus recreation offerings are not necessarily focused on chronic illness, so as a result, UC DPP would be competing for participants with other recreation programs. One UC DPP lead described the existence of “territories” and “fiefdoms,” and explained, “competing priorities, there’s so much going on in any one of the campuses.” According to one campus lead, “Sometimes, it competes with our other programs...Some people rotate through our programs because they are available and free.” Another reported that there are so many new programs, it can become overwhelming.

## Discussion

Our study describes factors that enhanced the implementation of the UC DPP Initiative across a state-wide network of 10 UC campuses with diverse characteristics (e.g., size, location, resources allocated to health and wellbeing, affiliation with a university medical center, etc.). The perspectives of our UC DPP leaders and campus leads were highly consistent; discrepancies mainly stemmed from the differential salience of factors at the system vs. campus level. For instance, UC DPP leaders focused on the DPP being evidence based; while effectiveness was also important for campus leads, their focus was primarily on adapting and enhancing the curriculum to maximize retention. Implementation partners identified 7 themes that facilitated DPP implementation on UC campuses. Several facilitators identified in this study are consistent with those reported in other workplace (e.g., [23] and community and health care organization [9, 24] delivery system settings. These include utilizing existing networks and strategies for communication, the availability of funding, having leadership support and dedicated staff, compatibility between DPP values and those of the delivery system setting (e.g., the UC system), and access to specialized information [9, 23, 24]. Barriers reported by implementation partners in this study are also consistent with those described in research from other delivery system settings, including limited cost, time, and resources to devote to DPP implementation [9].

The findings of this study also highlight factors that are unique or can be tailored specifically to university settings. These include utilizing established, internal channels of university communication (e.g., employee and campus listserves, newsletters, flyers, and mailers) to market UC DPP through multiple channels (e.g., Human Resources, campus health initiatives, and Recreation platforms) to reach a broad audience and target at-risk students, staff, and faculty. Other facilitators included convenience, accessibility, and the value of holding information and/or orientation sessions. Many campus leads reported enhancing the DPP curriculum with campus resources and events, and collaborated with professionals across campus disciplines to ensure that information was commensurate with the health literacy and educational level of participants. Implementation partners also underscored the critical importance of the UC system in providing funding and in-kind resources to offer UC DPP free of charge to participants, support the hiring of experienced lifestyle coaches (often from the School of Medicine or other health professions), allocate meeting space, and importantly, to establish and maintain the UC DPP Coordinating Center, which provides technical assistance, logistics, and coordination. Finally, campuses accessed established wellness linkages and resources (e.g., offering the program under a wellness or recreation umbrella, incorporating DPP into broad health-related campus initiatives and platforms, and being affiliated with a campus medical center).

Barriers to implementation were also noted. Some campuses have less extensive marketing and recruitment channels, fewer supplemental resources (e.g., to supplement the DPP curriculum, including guest speakers, teaching kitchens, paying for program materials, hiring coaches and ensuring staff stability, securing room reservations, and to generally support the sustainability of the program), and are less wellness focused, posing challenges to implementation. Campus leads also reported concerns about confidentiality and the time and resources required for data collection, incompatibility of sessions with participant schedules, and difficulties scheduling consistent locations for meetings.

While the diversity of UC campuses and affiliated partners advances our understanding of contributors to their success, there are limitations. Our findings may not generalize to all higher educational institutions or other workplace settings. However, our study includes the perspectives of campus leads from all 10 UC campuses which vary in geographic location, size and resources, as well as UC DPP leaders, who voiced their experiences with DPP across their diverse campus networks. There are also significant opportunities for future research that require additional work, including focusing on expanding reach to university students (e.g., use of social media platforms). Future research should also identify the benefits and challenges of having one’s workplace and healthcare at the same site, which offers numerous benefits but also confers concerns related to confidentiality.

Despite these limitations, this study offers the opportunity for interested universities to evaluate the factors impacting the implementation of DPP described here to better anticipate and problem-solve barriers to enhance program success. Our findings address an important gap in our understanding of facilitators and challenges concerning implementation of DPP in university settings and can serve as a preliminary roadmap for interested universities to consider when making decisions about the feasibility of adopting DPP on their campuses.

## Conclusion

This is one of the first studies to examine the challenges and opportunities of delivering the DPP intensive lifestyle change program across 10 university sites within the University of California system. Understanding implementation partners’ perceptions of the factors influencing success can be leveraged to enhance UC DPP efforts and help inform delivery strategies of other health and wellness programs across other university settings with similar characteristics.

## Supplementary Information

Supplementary Material 1.

## Data Availability

The datasets used and/or analyzed during the current study are available from the corresponding author on reasonable request.

## Abbreviations

UC DPP: University of California Diabetes Prevention Program
RE-AIM: Reach, effectiveness, adoption, implementation, and maintenance
UC: University of California
