# Supplementary material for: Implementation lessons learned from the University of California’s Diabetes Prevention Program Initiative
Source: BMC Public Health. 2024 Oct 11;24:2777. doi: 10.1186/s12889-024-20198-1 (PMC11468268; doi:10.1186/s12889-024-20198-1)
Supplement: Supplementary file 1 — Supplementary Material 1. [file 12889_2024_20198_MOESM1_ESM.docx]

Appendix 1: UC DPP leader interview guide

General

1. What is your role at UC [insert campus name] and what is your role or relationship with UC DPP?

2. How did you become involved with UC DPP?

3. What motivated you to get involved?

4. How long have you been involved with UC DPP? Has your role changed over time?

Reach

5. Has your campus done anything to raise awareness of prediabetes and UC DPP? If so, what?

6. What UC DPP outreach/recruitment strategies has your campus used?

7. Do you feel UC DPP participants reflect your campus population or are there subgroups that have not be willing or able to access the program?

8. Why do you think some faculty and staff participate in DPP?

9. Why do you think some faculty and staff decline participation in UC DPP?

10. What on-going or future efforts are you using to ensure a diverse representation of at-risk

individuals between students/faculty/administration and ethnicity/gender/etc.?

11. What are some ways to increase the reach of UC DPP on your campus or across UC? (e.g., for groups who may be less likely to participate)

Effectiveness

12. How do you evaluate the success of DPP on your campus? What outcomes are of value to you?

13. How effective would you say UC DPP has been on your campus?

14. What are the strengths of UC DPP?

15. What are some UC DPP areas that need improvement?

16. What feedback have you collected from UC DPP participants formally or informally? What do participants say?

17. I would like to get your opinion about specific program components, such as participant materials and reports your campus receives from the Coordinating center. If you are not familiar with a specific component, we can skip that question and move on to the next one. (some stakeholders may not have detailed knowledge about specific components)

a. Participant materials?

b. Lifestyle coach training hosted by the UC DPP Coordinating Center?

c. Overall quality of UC DPP coaches? (their own or the lifestyle coach training)

d. Number and timing of DPP sessions?

e. Data your campus collects for the UC DPP Coordinating Center?

f. Data your campus collects for the CDC?

g. Data reports your campus receives from the UC DPP Coordinating Center?

Adoption

18. What are facilitators to UC DPP adoption on your campus (things that made it easier to adopt UC DPP on your campus)?

19. What are barriers to UC DPP adoption on your campus (things that made it harder to adopt UC DPP on your campus)?

20. In what ways do you feel the UC [insert campus] community benefits from providing UC DPP on campus?

21. In what ways do you feel DPP participants benefit from the program?

22. If you were going to advise other universities planning adopt DPP, what would you recommend?

Implementation

23. Can you describe the UC DPP implementation process on your campus? [please describe the process of starting UC DPP on your campus]

24. Who have been the key UC DPP stakeholders on your campus? Probe for specific names

a. For example, we have heard about campuses working with HR or campus recreation

25. Have local campus leaders been involved in UC DPP?

a. If so, can you tell me more about those interactions?

26. Can you share some lessons learned during the implementation process?

27. What types of implementation challenges did you face or are you facing?

a. What worked or did not work with:

i. Participant recruitment?

ii. Participant engagement/retention?

28. Have you interacted with the UC DPP Coordinating Center?

a. If so, how often are you in touch with the UC DPP Coordinating Center?

b. Have these interactions been helpful?

c. In what areas would you like more support from the coordinating center?

29. Have you made adaptations to the UC DPP to meet your campus or participant needs?

30. Have you experienced any unintended consequences of UC DPP?

Maintenance

31. To what extent do you anticipate UC DPP will become established in your campus culture and workflow?

32. What are the obstacles to sustaining UC DPP on your campus over time?

33. If we were to continue offering UC DPP, what do you think you would need to maintain delivery on your campus?

34. Does your local leadership provide upkeep and necessary support for UC DPP maintenance?

a. Financial support?

b. In kind support? (e.g., free advertisement, use of rooms/meeting locations, staffing)

35. How well does the UC DPP fit in with the other specific wellness programs and interventions on your campus?

36. Does UC DPP help achieve your campus’s or UC core-objectives? If so, how? If not, why?

COVID

37. How have you balanced your involvement in UC DPP with campus priorities during pandemic?

38. How do you think COVID has impacted DPP on your campus?

39. Is there anything else you would like to share about your experience with UC DPP before we end this interview?

Appendix 2: UC DPP campus lead interview guide

General

1. What is your role at UC and what is your role or relationship with UC DPP?
2. How did you become involved with UC DPP?
3. What motivated you to get involved?
4. How long have you been involved with UC DPP? Has your role changed over time?

Reach

1. Has your office or team done anything to raise awareness of prediabetes and UC DPP? If so, what?
2. Do you feel UC DPP participants reflect the UC population or are there subgroups that have not be willing or able to access the program?
3. Why do you think some UC faculty and staff participate in UC DPP?
4. Why do you think some UC faculty and staff decline participation in UC DPP?
5. What on-going or future efforts are you using to ensure a diverse representation of at-risk individuals between students/faculty/administration and ethnicity/gender/etc.?
6. What are some ways to increase the reach of UC DPP across UC?

Effectiveness

1. How do you evaluate the success of DPP across UC? What outcomes are of value to you?
2. How effective would you say UC DPP has been overall?
3. What are the strengths of UC DPP?
4. What are some UC DPP areas that need improvement?
5. What feedback have you received about the program formally or informally from participants or campus teams?

Adoption

1. What are facilitators to UC DPP adoption across UC (things that made it easier to adopt UC DPP)?
2. What are barriers to UC DPP adoption across UC (things that made it harder to adopt UC DPP)?
3. In what ways do you feel the UC community benefits from providing UC DPP on campus?
4. In what ways do you feel DPP participants benefit from the program?
5. If you were going to advise other universities planning to adopt DPP, what would you recommend?

Implementation

1. Who have been the key UC DPP stakeholders across campuses?
   1. For example, we have heard about campuses working with HR or campus recreation.
2. Can you share some lessons learned during the UC DPP implementation process?
3. What types of implementation challenges did you face or are you facing? Or have you heard about any of these implementation challenges from your team?
   1. What worked or did not work with:
      1. Stakeholder engagement?
      2. Communication with individual campuses?
      3. Participant recruitment?
      4. Participant engagement/retention?
4. Have you interacted with the UC DPP Coordinating Center?
   1. If so, how often are you in touch with the UC DPP Coordinating Center?
   2. Have these interactions been helpful?
   3. In what areas would you like more support from the Coordinating center?
5. Have you made adaptations to the UC DPP to meet UC or participant needs?
6. Have you experienced any unintended consequences of UC DPP?

Maintenance

1. To what extent do you anticipate UC DPP will become established in UC culture and workflow?
2. What are the obstacles to sustaining UC DPP across UC over time?
3. What do you think UC campuses need to maintain UC DPP delivery over time?
4. Do you feel campus and UC leadership should provide upkeep and necessary support for UC DPP maintenance?
   1. Financial support?
   2. In kind support? (e.g., free advertisement, use of rooms/meeting locations, staffing)
5. How well does UC DPP fit in with the other specific wellness programs and interventions across UC?
6. Does UC DPP help achieve your UC core-objectives? If so, how? If not, why?

COVID

1. How have you balanced your involvement in UC DPP with campus priorities during pandemic?
2. How do you think COVID has impacted UC DPP?
3. Is there anything else you would like to share about your experience with UC DPP before we end this interview?
